# Microstructure and Mechanical Properties of Novel High-Strength, Low-Activation W_x_(TaVZr)_100−x_ (x = 5, 10, 15, 20, 25) Refractory High Entropy Alloys

**DOI:** 10.3390/e24101342

**Published:** 2022-09-23

**Authors:** Jingsai Zhang, Shunhua Chen, Jiaqin Liu, Zhenhua Qing, Yucheng Wu

**Affiliations:** 1 School of Mechanical Engineering, Hefei University of Technology, Hefei 230009, China; 2 National-Local Joint Engineering Research Centre of Nonferrous Metals and Processing Technology, Hefei 230009, China; 3 School of Materials Science and Engineering, Hefei University of Technology, Hefei 230009, China

**Keywords:** refractory high entropy alloy, microstructure, strength, low-activation, fracture

## Abstract

In this work, novel high-strength, low-activation W_x_(TaVZr)_100−x_ (x = 5, 10, 15, 20, 25) refractory high entropy alloys (RHEAs) were prepared by vacuum arc melting. Their microstructure, compressive mechanical properties, hardness, and fracture morphology were investigated and analyzed. The results show that the RHEAs possess a disordered BCC phase, ordered Laves phase, and Zr-rich HCP phase. Their dendrite structures were observed, and the distribution of dendrites became gradually more dense with an increase in W content. The RHEAs demonstrate high strength and hardness, with these properties being higher than in most reported tungsten-containing RHEAs. For example, the typical W_20_(TaVZr)_80_ RHEA has a yield strength of 1985 MPa and a hardness of 636 *HV*, respectively. The improvement in terms of strength and hardness are mainly due to solid solution strengthening and the increase in dendritic regions. During compression, with the increase in the applied load, the fracture behavior of RHEAs changed from initial intergranular fractures to a mixed mode combining both intergranular and transgranular fractures.

## 1. Introduction

In recent years, a new type of alloy, noted as refractory high entropy alloy (RHEA), has great application potential for nuclear plant applications. RHEAs are designed by introducing refractory elements (such as W, Nb, Mo, Ta, Ti, V, Cr, Zr, and Hf), which endowed RHEAs with more properties than HEAs, such as a high strength [1,2,3], high plasticity [4,5,6], strong oxidation resistance [7,8,9], corrosion resistance [10,11], radiation resistance [12,13,14], and wear resistance [15,16]. RHEAs have therefore attracted extensive research attention due to their unique structures and excellent performances. For example, Senkov et al. [1] took the lead in designing and preparing WNbMoTa and WNbMoTaV RHEAs with yield strengths of 1058 MPa and 1246 MPa at room temperature (RT), respectively. Since the limited RT plasticity of these two RHEAs restricts their engineering applications, Senkov and his coworkers then developed a ductile TaNbHfZrTi RHEA with plastic strain ε ≥ 50% and yield strength of 929 MPa [6]. In order to further improve the strength, Juan et al. [17] designed and synthesized HfMoTaTiZr and HfMoNbTaTiZr RHEAs based on the TaNbHfZrTi RHEA, where a yield strength of 1600 MPa and 1512 MPa were achieved, respectively. Moreover, the HfMoNbTaTiZr RHEA still retained a fracture strain of 12%. In addition to the excellent mechanical properties at RT, RHEAs also have excellent mechanical properties at high temperature (HT). For example, the WNbMoTa and WNbMoTaV RHEAs can maintain high yield strength of 405 MPa and 477 MPa at 1600 °C, respectively. HfMoTaTiZr and HfMoNbTaTiZr RHEAs also exhibited yield strengths of 404 MPa and 556 MPa, respectively, at 1200 °C. With favorable combinations of remarkable properties, RHEAs are regarded as potential candidates for high-temperature applications in the aerospace, military, and nuclear industries.

Research on low-activation RHEAs is conducive to promoting the development of Gen-IV nuclear fission and fusion reactors [18,19,20,21]. However, most RHEA systems contain high activation elements, such as Mo and Nb. Such classes of RHEAs cannot be used as structural materials for nuclear energy or other radioactive fields due to their vulnerability to radiation damage. Moreover, high activation elements take a long time to reach the hands-on standard after the decommissioning of relevant equipment, for example, ~10^4^ and ~3 × 10^5^ years for Mo and Nb, respectively [19,22]. Low activation elements, such as W, Ta, Ti, V, Zr, Cr, Fe, and Mn [19,21], can fundamentally avoid this problem, because the time required for most low activation elements to reach the “hands-on level” is less than 100 years. And RHEA, which features low activation elements as its constituent elements, also has excellent mechanical properties at RT and HT. For example, Ma et al. [23] synthesized a novel low-activation WTaHfTiZr RHEA, and the compression test result showed that the yield strength and fracture strain at RT are 1900 MPa and 8.1%, respectively. Moreover, the alloy still possesses a yield strength of 203 MPa at 1000 °C, which make it possible for it to be considered as a preferred material for engineering applications in the field of nuclear energy, such as in a nuclear reactor.

Although the development of low-activation RHEAs has been reported [18,19,20,21,23,24,25,26], most low-activation RHEA systems contain non-refractory or high-activation elements. It is still challenging to develop RHEAs consisting of all refractory and low-activation elements. Moreover, the mechanical properties of the reported low-activation RHEAs have not been thoroughly studied, including the effect of changing elements on their microstructures and mechanical properties. For example, although Ayyagari et al. [19] reported a low activation RHEA consisting of all refractory elements (TiVZrTaW), only the microstructure and the hardness at different temperatures were presented. Therefore, it is still important to develop high-performance RHEAs consisting of all refractory and low-activation elements and to study the effect of compositional changes on their microstructures and mechanical properties in depth. For this purpose, four refractory elements, W, Ta, V, and Zr, all with low activation, were selected as the constituent elements to design new RHEA systems in this work. Among them, W has the highest melting point of metals (*T*_m_ = 3695 K), which is also considered as an ideal element for nuclear structural materials [27,28,29,30]. The Ta element possesses a small thermal expansion coefficient and similar chemical properties to W. The addition of low-density V (*ρ*_V_ = 6.110 g/cm^3^) and Zr (*ρ*_Zr_ = 6.511 g/cm^3^) elements can ensure that the designed RHEAs have a high strength and lower density at the same time. Moreover, the addition of Zr can form an excessive atomic size difference (*δ*) with other elements and cause lattice distortion due to its large atomic radius, which can promote the formation of intermetallic compounds, resulting in the enhancement of mechanical properties [31]. Based on above, novel W_x_(TaVZr)_100−x_ RHEAs were designed and prepared. The effects of various W contents on their microstructural characteristics and compressive mechanical properties were systematically investigated. The underlying mechanisms for the phase formation and strengthening of the RHEAs were also examined and discussed.

## 2. Materials and Methods

The bulk raw materials of W, Ta, V, and Zr elements, with purities higher than 99.95%, were used to fabricate W_x_(TaVZr)_100−x_ RHEAs (x = 5, 10, 15, 20, and 25, denoted as W5, W10, W15, W20, and W25, respectively). The RHEA ingots were prepared by vacuum arc melting under a Ti-gettered, high-purity argon atmosphere. The ingots were remelted 12 times to ensure homogeneity, where, in each melting, the ingot was flipped and was in the flow state for 5 min. Each ingot was approximately 40 g and the mass loss of an arc-melted specimen was less than 0.1 g. The phase structures of the RHEAs were analyzed by a PANalytical X-Pert PRO MPD X-ray diffractometer (XRD), with the 2*θ* scanning angle ranging from 10° to 120°. The microstructures of the RHEAs were characterized by field emission scanning electron microscopy (FESEM, Hitachi SU8020, Japan), and their element distributions were analyzed by the equipped energy dispersive spectrometer (EDS). The specimens were polished carefully for SEM observations by SiC abrasive papers with grits reaching to 5000. Cuboid specimens with sizes of 3 mm × 3 mm × 6 mm were cut from the ingots using wire-cut electrical discharge machining (WEDM). RT compression tests of the RHEAs were performed on an electronic universal mechanical testing machine at an initial strain rate of 5 × 10^−4^ s^−1^. After compression tests, their fracture features were inspected by SEM observations on a JSM-6490LV scanning electron microscope. The Vickers hardness was measured on polished surfaces using an HV-1000SA Vickers hardness tester under 1000 gf load applied for 10 s.

## 3. Results and Discussion

### 3.1. Phase Analysis

During the past few decades, some empirical parameters have been proposed to predict the phase formation and structural stability of HEAs. Based on the “Hume-Rothery” criterion, Zhang et al. [32] studied the influence of Δ*S*_mix_, the enthalpy of mixing (Δ*H*_mix_), and *δ* on the formation of the solid solution phase of HEAs, and the parameter ranges for the formation of the solid solution phase in HEAs was summarized as: 12 J/(K·mol) ≤ Δ*S*_mix_ ≤ 17.5 J/(K·mol), −15 kJ/mol ≤ Δ*H*_mix_ ≤ 5 kJ/mol, and *δ* ≤ 6.6%.

Studies [32,33,34] have further expounded the function of Δ*S*_mix_, Δ*H*_mix_, and *δ* on phase formation in HEAs. A high Δ*S*_mix_ value can increase the compatibility between principal components, reduce the trend of ordering and segregation, and then avoid the formation of intermetallic compounds or other ordered phases. The closer the Δ*H*_mix_ value is to zero, the easier the solid solution structure can form in HEAs. The parameter *δ* was proposed to characterize the stability of solid solution, where a larger *δ* value tends to result in a lower stability in terms of the solid solution. These three parameters are defined by the expressions:

(1) $$\Delta S_{\mathrm{mix}}=-R\overset{n}{\underset{i=1}{\sum }}c_{i}\ln c_{i}$$

(2) $$\Delta H_{\mathrm{mix}}=\overset{n}{\underset{i=1,\;i\neq j}{\sum }}4\Delta H_{\mathrm{ij}}^{\mathrm{mix}}c_{i}c_{j}$$

(3) δ=∑i=1nci(1−rir¯)2

where *c*_i_ and *c*_j_ are the atomic percentages of the *i*th and *j*th elements, respectively, *R* (= 8.3144 J/(K·mol)) is the gas constant, $\Delta H_{\mathrm{ij}}^{\mathrm{mix}}$ represents the enthalpy of the mixing of the binary liquid i-j alloy that can be obtained based on the Miedema’s model [35], *r*_i_ is the atomic radius of the *i*th element, $\bar{r}=\overset{n}{\underset{i=1}{\sum }}c_{i}r_{i}$ is the average atomic radius, and n is the number of alloy elements.

In order to accurately predict the formation of solid solution phase, Yang et al. [33] further proposed a new parameter, Ω, from the perspective of free energy, to comprehensively consider the influence of Δ*S*_mix_ and Δ*H*_mix_ on the formation of solid solution phase. The Ω-*δ* criteria was proposed in combination with the parameter *δ*, with the idea being that HEAs are more likely to form solid solution phase when the two parameters fulfil the following ranges: Ω ≥ 1.1 and *δ* ≤ 6.6%. The parameter Ω can be calculated as:

(4) $$\Omega =\frac{T_{m}\Delta S_{\mathrm{mix}}}{\left|\Delta H_{\mathrm{mix}}\right|}$$

*T*_m_ is the melting temperature of the alloy, which can be calculated using the rule of mixtures:

(5) $$\;T_{m}=\overset{n}{\underset{i=1}{\sum }}c_{i}{\left(T_{m}\right)}_{i}$$

Here, (*T*_m_)_i_ is the melting temperature of the *i*th element.

The above parameters can only judge whether the alloy can be noted as “HEA” or whether the alloy can form solid solution phase. They cannot determine the structure of solid solution phase. According to the “Hume-Rothery” criterion, Guo et al. [36] proposed the parameter valence electron concentration (*VEC*) to predict the structure of solid solution phase in HEAs: a single BCC or FCC solid solution phase tends to form when *VEC* < 6.87 or *VEC* ≥ 8.0, respectively, whereas BCC and FCC solid solution phases tend to coexist when 6.87 ≤ *VEC <* 8.0. Such a parameter can be obtained using the rule of mixture:

(6) $$VEC=\overset{n}{\underset{i=1}{\sum }}c_{i}{\left(VEC\right)}_{i}$$

where (*VEC*)_i_ is the *VEC* of element *i*.

In this work, the relevant parameters of the constituent elements for the designed W_x_(TaVZr)_100−x_ RHEAs are shown in Table 1. The empirical parameter values of the RHEAs calculated according to Equations (1)~(6) are listed in Table 2. It can be seen that the Δ*S*_mix_ values of the RHEAs range from 10.328 to 11.526 J/(K·mol), which are not within the range of 12~17.5 J/(K·mol). However, the Δ*S*_mix_ values are far higher than that of most traditional alloys. Relatively high Δ*S*_mix_ values can make the disordered solid solution phase more stable than ordered intermetallic compounds [37]. The Δ*H*_mix_ values of the W_x_(TaVZr)_100−x_ RHEAs are in the range of −15~5 kJ/mol, and the *VEC* values are smaller than 6.87, indicating that the RHEAs will form BCC solid solution phase. The Ω values are much larger than 1.1, whereas the *δ* values are larger than 6.6%. The relevant study [33] showed that with a relatively large *δ* value, the system tends to form intermetallic compounds. Thus, the present W_x_(TaVZr)_100−x_ RHEAs may form BCC solid solution phase, accompanied by intermetallic compounds.

**Figure 1:**
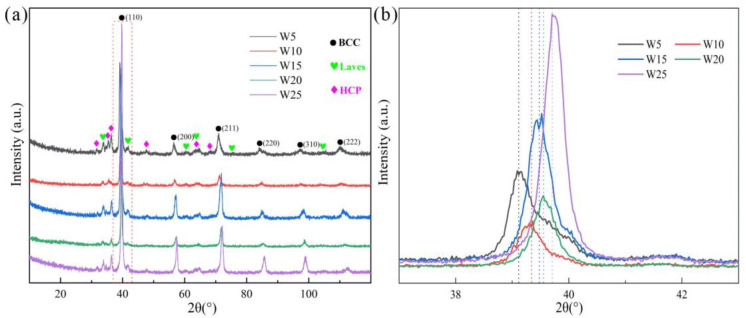
XRD patterns of the W_x_(TaVZr)_100−x_ RHEAs (**a**), where (**b**) shows the magnified (110) peaks in (**a**).

The XRD patterns of the RHEAs are depicted in Figure 1. It can be seen that the RHEAs are not single-phase but have multi-phase structures. Further analysis has shown that the RHEAs consist of three phases: the main BCC phase, Laves phase (V_2_Zr), and Zr-rich HCP phase. By amplifying the (110) diffraction peak of the BCC phase (Figure 1b), it can be seen that with the increase in W content, the diffraction peak gradually moved to a higher diffraction angle, indicating the occurrence of lattice distortion. The experimental and theoretical lattice constants of the BCC phase are listed in Table 3. The results indicate that the lattice constant of the BCC phase decreased gradually with the increase in W content. This may be caused by the reduction of the total atomic radius of the RHEAs due to the introduction of more W elements with a smaller atomic radius. This result is consistent with the result that the (110) diffraction peak shifted to the higher diffraction angle, as shown in Figure 1b. Intriguingly, both the experimental and theoretical lattice constant values exhibited similar decreasing trends. The theoretical values are slightly larger than the experimental values, which suggests that some ordered phases may be formed in these RHEAs [42]. This result can also verify the existence of Laves phase. Based on the rule of mixture, the theoretical lattice constant, *a*_mix_, of RHEAs can be estimated as [42]:

(7) $$a_{\mathrm{mix}}=\overset{n}{\underset{i=1}{\sum }}c_{i}a_{i}$$

where *a*_i_ is the lattice constant of the *i*th element. Yeh et al. indicated that the increase in the lattice constant may result from the corresponding large lattice strain effect [34]. The shift of diffraction peak in this work may also be related to lattice distortion, where qualitatively analysis can be conducted. Although the lattice distortion magnitudes can be quantitative characterized by the XRD diffraction peak [43,44], the results are also affected by the crystal texture, atomic fluorescence effect, instrument errors, and testing environment [45]. For example, Owen et al. examined the effect of such factors on diffraction peaks, and the lattice distortion was measured by a more accurate approach where the total scattering data were collected in an irradiation environment [45]. In this work, since the decrease in lattice constants for the BCC phase was clearly observed from both the experimental and theoretical calculations, a detailed characterization of lattice distortion was not conducted here.

### 3.2. Microstructure and Chemical Compositions

**Figure 2:**
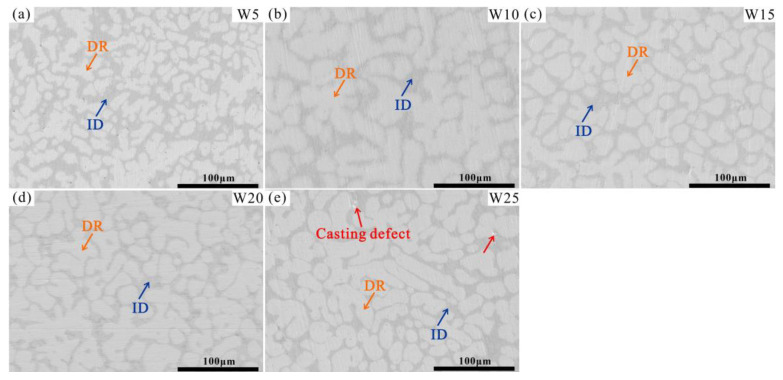
SEM images of the W_x_(TaVZr)_100−x_ RHEAs, where (**a**–**e**) show the W5, W10, W15, W20, and W25 RHEAs, respectively.

**Figure 3:**
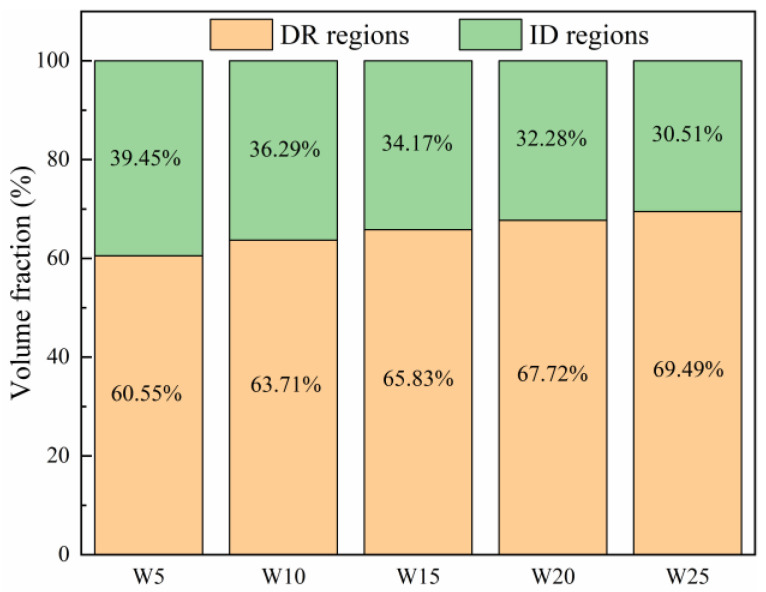
The volume fractions of DR and ID regions of the W_x_(TaVZr)_100−x_ RHEAs.

**Figure 4:**
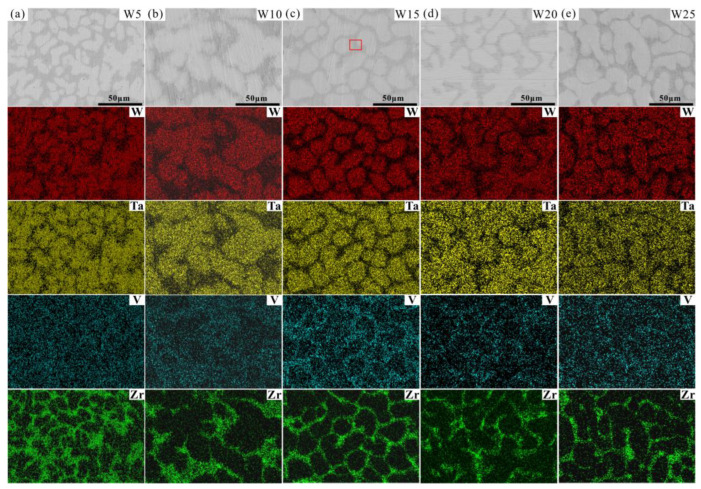
EDS mapping results showing the element distributions of the W_x_(TaVZr)_100−x_ RHEAs: (**a**) W5, (**b**) W10, (**c**) W15, (**d**) W20 and (**e**) W25.

The microstructures of the W_x_(TaVZr)_100−x_ RHEAs are shown in Figure 2. The SEM images of the RHEAs mainly show two regions: white regions and gray regions, representing dendritic regions (DR) and interdendritic regions (ID), respectively. The microstructures of RHEAs changed significantly with the increase in W content. W5 RHEA mainly manifested slender dendrites, and most of them were small-sized dendrites (Figure 2a). With the increase in W content, the dendrite shape of the W10 RHEA became coarser, and the dendrite size also increased (Figure 2b). Moreover, for these two RHEAs, the distribution of dendrite structure was loose, leaving more interdendrites. With a further increase in W content, the microstructures of the W15, W20, and W25 RHEAs were mainly composed of dendrites of different sizes, and the distribution of dendrites was denser when compared with the W5 and W10 RHEAs, as shown in Figure 2c–e. The Image J software was further used to calculate the volume fractions of ID regions, where 39.45%, 36.29%, 34.17%, 32.28%, and 30.51% for W5, W10, W15, W20 and W25, respectively, were observed, as shown in Figure 3. The element distribution results for RHEAs (Figure 4) show that the W and Ta elements were mainly concentrated in DR regions, while the V and Zr elements were mainly concentrated in ID regions. The results indicate the occurrence of element segregation, which is common in as-cast RHEAs [4,46]. The main reason for this could be that during the solidification process, elements with a high melting point tend to solidify first to form DR regions, while and elements with a low melting point will then solidify to form ID regions [46].

**Figure 5:**
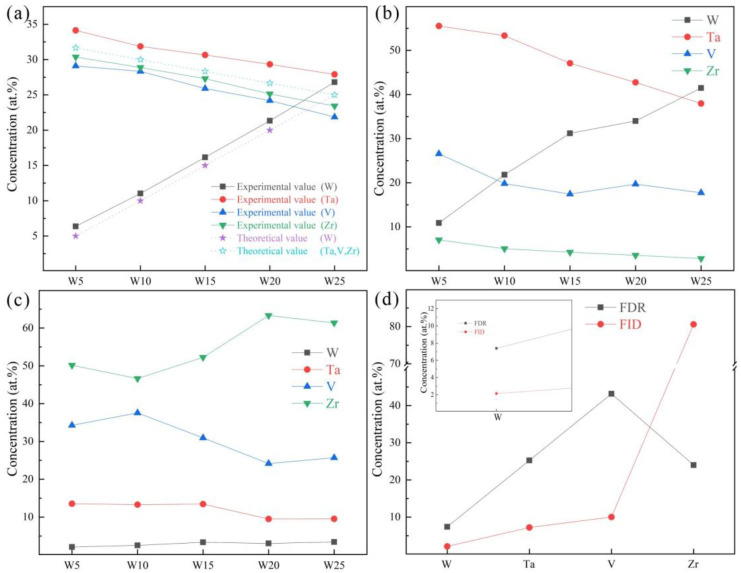
The chemical compositions of prepared RHEAs: (**a**) mapping results; (**b**) point scanning in dendritic regions (DR); (**c**) point scanning in interdendritic regions (ID); and (**d**) point scanning in the fine dendritic regions (FDR) and fine interdendritic regions (FID) for the W15 RHEA.

The chemical compositions of the RHEAs are given in Figure 5a, and their detailed chemical compositions are listed in Table 4. It can be seen that the W and Ta contents were slightly higher than the theoretical values, while the V and Zr contents were slightly lower than the theoretical values. This may be caused by element segregation during the solidification process, where the solidification sequence of different elements is distinct, resulting in different element contents at different positions in the RHEA ingots. The slight volatilization of low melting point elements (V and Zr) during the melting of RHEAs may also be responsible for this result. Furthermore, the chemical compositions of DR and ID regions are depicted in Figure 5b,c. For the W5, W10, and W15 RHEAs, the W content in the DR region is about twice its theoretical value.

**Figure 6:**
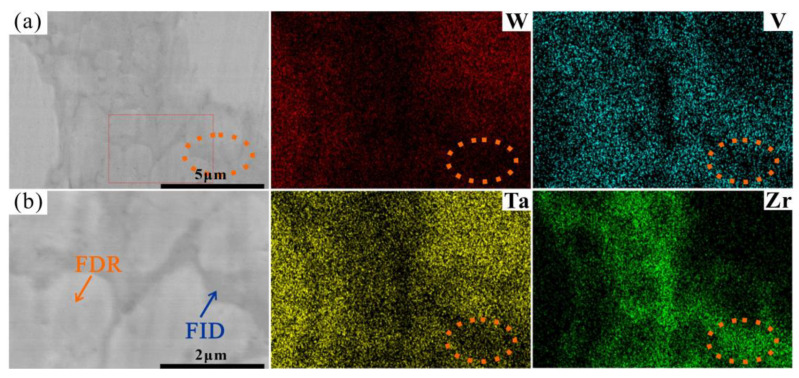
EDS mapping results showing distributions of W, V Ta, and Zr elements at the area indicated by the red rectangle in Figure 4c, where (**b**) shows the rectangles in (**a**) at a higher magnification.

More interestingly, it can be seen from the SEM images in Figure 2 and Figure 4 that the ID region of the RHEAs appears to be divided into two different parts, which can be confirmed by the high magnification images of the W15 RHEA in Figure 6. The two parts were named as fine dendritic regions (FDR) and fine interdendritic regions (FID) in this work, and the corresponding EDS results are also shown in Figure 6. The V and Zr elements were mainly accumulated in FDR regions and the Zr element in FID regions. The point scanning results are given in Figure 5d, and the findings are consistent with the EDS mapping results. Moreover, the content ratio of V to Zr elements in FDR regions is approximately 1.799, and the Zr content in FID regions is as high as 80.61%. According to the present XRD results and the findings in a previous study [47], it can be deduced that the RHEAs could have V_2_Zr Laves phase and Zr-rich HCP phase in addition to the main BCC phase. Therefore, the BCC phase was mainly concentrated in the DR region, while the Laves and Zr-rich HCP phases were mainly concentrated in the ID region. The Laves phase enriched with V and Zr was mainly gathered in FDR regions, and the HCP phase highly enriched with Zr was concentrated in FID regions. The main reason for the formation of Laves phase could be attributed to the largest difference in terms of atomic radius between V and Zr elements among the four constituent elements (Table 1). A larger difference between elements in terms of atomic radius can cause a stronger binding force between elements, which may lead the formation of Laves phase. The HCP lattice structure of the Zr element concentrated in ID or FID regions may promote the formation of Zr-rich HCP phase in these regions.

### 3.3. Mechanical Properties

**Figure 7:**
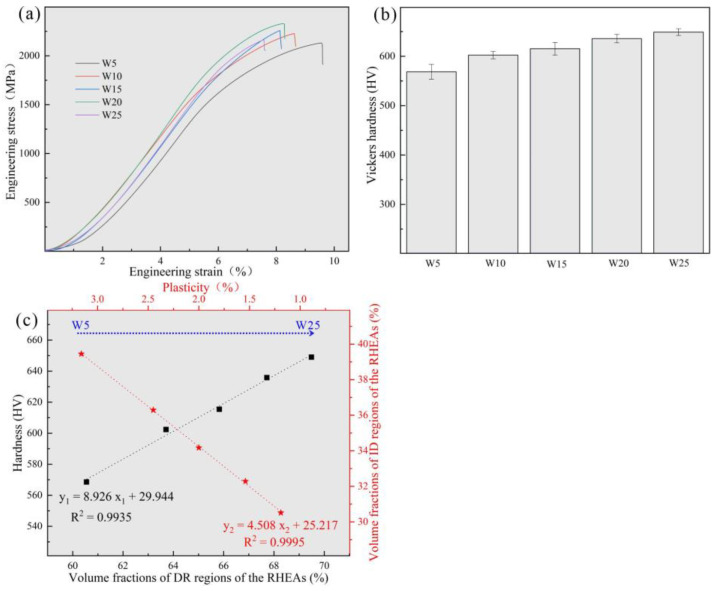
(**a**) Compressive engineering stress–strain curves at RT; (**b**) Vickers microhardness of W_x_(TaVZr)_100−x_ RHEAs; and (**c**) the relationships among the hardness and volume fractions of DR regions, plasticity, and the volume fractions of ID regions.

The RT compressive stress–strain curves of the RHEAs are shown in Figure 7a, and their detailed mechanical properties are listed in Table 5. With the increase in W content from 5 at.% to 20 at.%, the yield strength of the RHEAs gradually increased, reaching a maximum value of 1985 MPa in the W20 RHEA, which is larger than for most reported tungsten-containing RHEAs. The hardness value also exhibited the same increasing trend, as shown in Figure 7b. The improvement in terms of strength and hardness may be ascribed to the two factors. On the one hand, serious lattice distortion enhanced the solid solution strengthening effect of the RHEAs, which further increased the strength and hardness of the RHEAs. On the other hand, with the increase in W content, the volume fractions of DR regions increased (Figure 3). According to Figure 4 and Figure 5, the W and Ta elements mainly concentrated in DR regions resulted in the DR regions having a higher strength and hardness compared to the ID regions enriched with V and Zr elements. Therefore, with this overall increase in terms of strength and hardness, the W_x_(TaVZr)_100−x_ RHEAs have the potential to be used as wear-resistant material in engineering applications. The relationship between the volume fractions of DR regions and hardness is shown in Figure 7c. When the W content increased to 25 at.%, the strength of W25 RHEA decreased. Despite the differences in terms of lattice distortion between the two alloys, the present findings show that the decrease in terms of strength for the W25 RHEA may be attributed to the weak fluidity and the existence of casting defects (Figure 2e). The excessive W content in the W25 RHEA can reduce the fluidity of the alloy during the melting process, resulting in casting defects in the subsequent solidification process [48]. In contract to the RHEAs’ strength or hardness properties, with the increase in W content, the plasticity of the RHEAs showed a decreasing trend (Figure 7c). This may be caused by the attenuation of the volume fractions of the ID regions, where slip movement may occur during compression.

**Figure 8:**
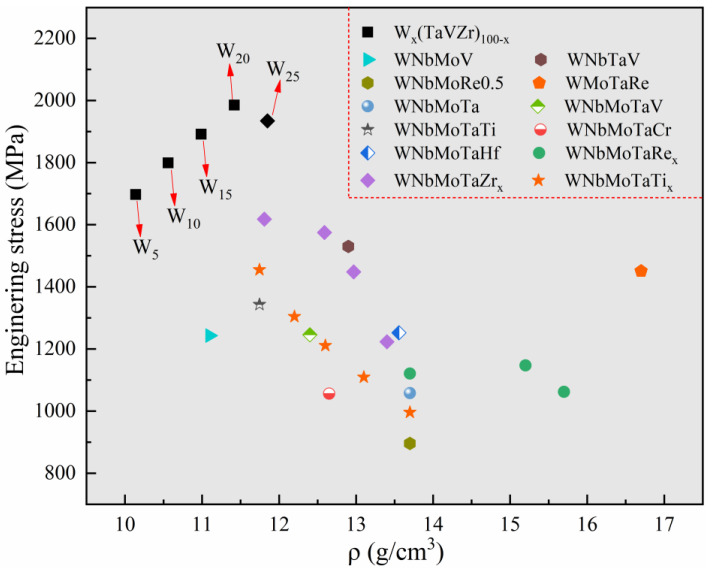
Schematic diagram showing a comparison in terms of strength and density between the designed RHEAs and some typical RHEAs in the literature, including WNbMoV [49], WnbTaV [46], WnbMoRe_0.5_ [50], WmoTaRe [51], WnbMoTa and WnbMoTaV [1], WnbMoTaTi [2], WnbMoTaCr and WnbMoTaHf [52], WnbMNoTaRe_x_ (x = 0, 0.5 and 1) [42], WnbMoTaZr_x_ (x = 0.1, 0.3, 0.5 and 1.0) [31], and WNbMoTaTi_x_ (x = 0, 0.25, 0.5, 0.75 and1) [53].

In order to compare the mechanical properties of the designed W_x_(TaVZr)_100−x_ RHEAs with other RHEAs in the literature, the mechanical properties and densities of tungsten-containing quaternary RHEAs [46,49,50,51], typical WNbMoTa RHEA [1], WNbMoTa-X (X represents other metallic elements) RHEAs [1,2,31,42,52,53], and the present RHEAs are given in Figure 8. The theoretical density of RHEAs can be obtained using the rule of mixture and expressed by following equation:

(8) $$\rho =\frac{\sum_{i=1}^{n}c_{i}A_{i}}{\sum_{i=1}^{n}\frac{c_{i}A_{i}}{\rho_{i}}}$$

where *A_i_* and *ρ_i_* are the atomic weight and density of the *i*th element, respectively. The calculated results of the designed RHEAs are also listed in Table 5. It can be seen that the present RHEAs not only have a relatively high strength but also maintain lower densities. Moreover, the constituent elements of the present RHEAs are all low activation elements [19], which may have the potential to be applied in the field of nuclear energy.

### 3.4. Fracture Behavior and Morphology

**Figure 9:**
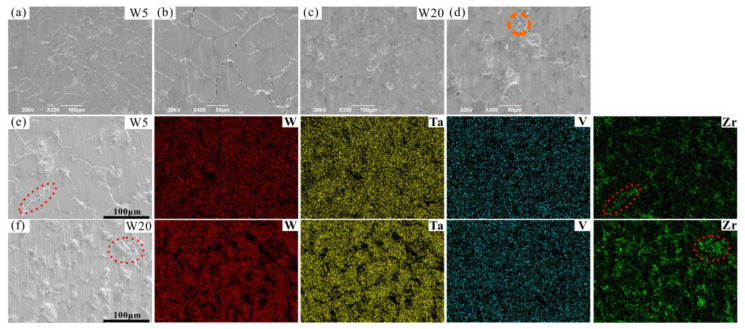
SEM images of the surface of RHEAs after compression tests, where (**a**–**d**) are the W5 and W20 RHEAs, respectively. The corresponding element distributions of W5 and W20 RHEAs at relatively higher magnifications are shown in (**e**,**f**), respectively.

SEM images of the surfaces of typical W5 and W20 RHEAs after compression tests are shown in Figure 9. The RHEAs with different W contents showed different surface morphologies after compression. For the W5 RHEA, microcracks formed in both ID and DR regions (Figure 9a,b). Meanwhile, with the increasing W content, microcracks mainly distributed in the ID regions for the W20 RHEA (Figure 9c,d). This may be due to the different microstructures of the two RHEAs, where microcracks mainly formed in the ID regions initially. As can be seen from Figure 9b, microcracks mainly appeared in ID regions. These microcracks were longer and more integrated than those in DR regions, indicating that the microcracks may emerge first in these regions during compression. The formation of microcracks in the ID regions of the W20 RHEA could be further validated. The formation of microcracks may be attributed to the formation of complex local stress fields within RHEAs, resulting from both the uneven distribution of elements and the lattice distortion effect [31,54]. As can be seen from Figure 9d, the molten droplet appeared in the ID region, as circled by the orange dotted line, which is usually observed on the fracture surface of bulk metallic glasses (BMGs) [55]. An EDS analysis of the surfaces of the W5 and W20 RHEAs after compression tests are shown in Figure 9e,f. The results show that the regions around the cracks were mainly enriched with the Zr element. Combined with the EDS results in Figure 4, which show that the Zr element was mainly concentrated in ID regions, it can be further proved that microcracks mainly occurred in ID regions. On the other hand, the formation of fewer cracks in the W20 RHEA should also due to the limited plasticity of the alloy. According to Figure 7a, the W20 alloy has a higher strength and lower plasticity than the W5 alloy, demonstrating more brittleness. The intrinsic brittleness of the W20 alloy may also be a potential reason for the morphology on the deformed surface, where the specimen fractured before the formation of more cracks within DR regions.

**Figure 10:**
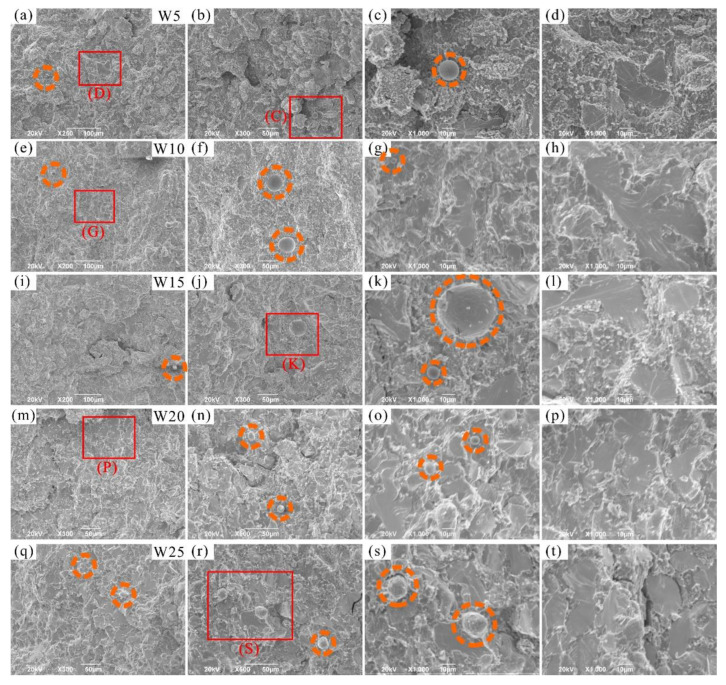
SEM images showing the fracture morphology of the prepared RHEAs after compression tests: (**a**–**d**) W5; (**e**–**h**) W10; (**i**–**l**) W15; (**m**–**p**) W20, and (**q**–**t**) W25, where (**c**,**d**,**g**,**k**,**p**,**s**) are the magnified images of the red rectangles C, D, G, K, P, and S, respectively.

**Figure 11:**
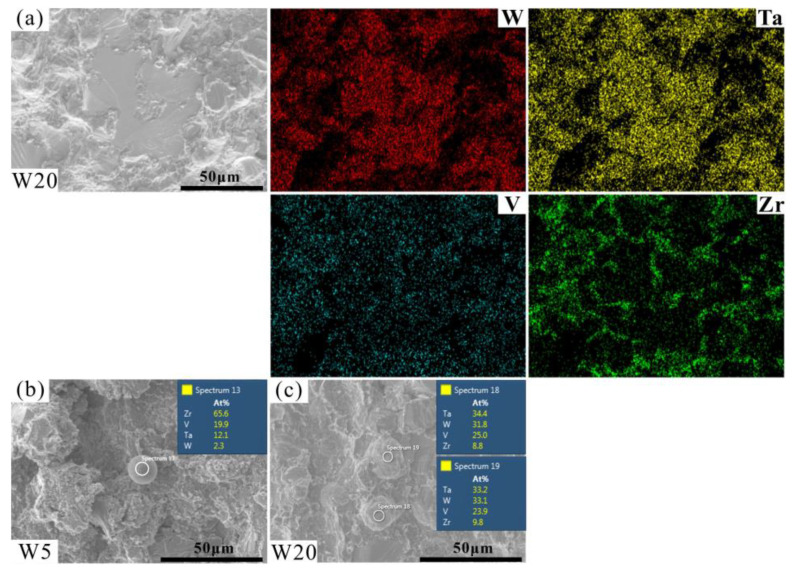
EDS mapping results showing the elemental distributions of the fracture surface of W20 RHEA (**a**) and the chemical composition of the molten droplets on the fracture surface, where (**b**,**c**) are W5 and W20 RHEAs, respectively.

The fracture morphologies of the RHEAs are shown in Figure 10. The fracture surfaces showed obvious river patterns and smooth planes surrounded by many particles, which are typical fracture characteristics normally apparent in brittle materials [56,57,58]. With the increase in W content, the area of river patterns and smooth planes increased gradually. Combined with the above-mentioned results, it can be inferred that these characteristics may be caused by the fracture of DR regions. This prediction can be further confirmed by the EDS results of the fracture surface of typical W20 RHEA, as shown in Figure 11a. The formation of river patterns and smooth planes indicated the appearance of transgranular fractures in these RHEAs, where the interdendrites could not bear the increasing loads. Therefore, the microcracks formed in ID regions extended to the DR regions and finally caused the fracture of dendrites. At the same time, the fracture mode of the RHEAs also changed from initial intergranular fractures to the mixed mode combining both intergranular and transgranular fractures. Further observations showed that molten droplets with different sizes also formed in the ID regions where fine particles gathered, as circled by the orange dotted line in Figure 10. The findings are consistent with the SEM images shown in Figure 9d. Senkov et al. [59] declared that the generation of particles was caused by explosive-like fractures during compression, which led to the fragmentation of the surrounding regions. A large amount of elastic energy is also released instantly upon fracturing, resulting in the melting and re-solidification of the material in nearby regions and the subsequent formation of droplets [59]. In this work, the appearance of debris and droplets on the fracture surface may also have been caused by the instant release of elastic energy. However, more effort should be devoted to the calculation and verification of such a prediction in future in order to reveal the underlying mechanisms.

Furthermore, the point scanning results show that the element contents of the molten droplets of RHEAs with varying W contents were different. For example, the W and Ta contents of the molten droplet of the W5 RHEA were lower than the V and Zr contents (as shown in Figure 11b), which is similar to the case of the W and Ta contents in ID regions (Figure 5c and Table 4). However, the W and Ta contents of the molten droplets of W20 RHEA were higher than the V and Zr contents (Figure 11c). Such a result may be caused by the gasification of V and Zr elements with low melting points and boiling points due to the large amount of energy released at fracture. To sum up, a conclusion can be drawn that during the compression process, with the increase in the applied load, the microcracks that appeared initially in interdendrites gradually extended into dendrites and eventually led to the failure of the RHEAs. Moreover, the fracture mode for the RHEAs also evolved from initial intergranular fractures to mixed fractures combining both intergranular and transgranular fractures.

## 4. Conclusions

In this work, novel high-strength, low-activation W_x_(TaVZr)_100−x_ RHEAs were designed and prepared. The effect of various W contents on the microstructures and mechanical properties of the RHEAs were explored. The phase formation, strengthening mechanisms, and fracture behavior were also analyzed and discussed. The main findings of the present work are summarized as follows:

(1) The prepared RHEAs have three different phases. In addition to the main disordered BCC solid solution phase, Laves and Zr-rich HCP phases were also detected. Through detailed analysis, it was confirmed that the Laves phase is an intermetallic compound (V_2_Zr) formed by V and Zr elements.

(2) With an increase in W content, the microstructure of the RHEAs changed significantly, from a slender dendrite shape to a coarser dendrite shape, and the distribution of dendrites continuously became denser. The W and Ta elements with high melting points were mainly concentrated in dendritic regions, while the V and Zr elements with low melting points were concentrated in interdendritic regions. In addition, the interdendritic regions were divided into two parts, with the fine dendritic regions being enriched with V and Zr elements and the fine interdendritic regions enriched with Zr element.

(3) With the increase in W content, the RHEAs displayed increased strength and hardness, which can be attributed to the effect of solid solution strengthening and the increase in dendritic regions.

(4) Microcracks initially formed in interdendritic regions during compression, and molten droplets also formed in interdendritic regions on the fracture surface due to the release of elastic energy at the fracture. With an increase in the applied load, the fracture mode of the RHEAs changed from initial intergranular fractures to a mixed fracture mode featuring the coexistence of both intergranular and transgranular fractures.

## Figures and Tables

**Table 1:** The relevant parameters for the constituent elements of W_x_(TaVZr)_100−x_ RHEAs [38,39,40,41].

Element	W	Ta	V	Zr
*r* (Å)	1.367	1.430	1.316	1.603
*VEC*	6	5	5	4
*a* (Å)	3.165	3.303	3.028	3.582
*T*_m_ (K)	3695	3290	2183	2128
* ρ * (g/cm^3^)	19.250	16.650	6.110	6.511
*HV* (kgf/mm^2^)	350	89	64	92

**Table 2:** The calculated empirical parameter values and melting point of W_x_(TaVZr)_100−x_ RHEAs.

Alloy	Δ*S*mix [J/(K·mol)]	Δ*H*mix (kJ/mol)	*δ* (%)	*VEC*	Ω	*T*m (K)
W5	10.328	−1.879	7.857	5.05	14.25	2591.73
W10	10.924	−2.760	7.758	5.10	10.49	2649.80
W15	11.279	−3.532	7.647	5.15	8.65	2707.88
W20	11.468	−4.196	7.523	5.20	7.56	2765.93
W25	11.526	−4.750	7.384	5.25	6.58	2824.00

**Table 3:** Experimental and theoretic lattice constants for the BCC phase of the W_x_(TaVZr)_100−x_ RHEAs.

Alloy	Lattice Constant (Å)	
	Experimental	Theoretic
W5	3.2541	3.2974
W10	3.2388	3.2904
W15	3.2261	3.2835
W20	3.2197	3.2765
W25	3.2077	3.2695

**Table 4:** The detailed chemical compositions of arc-melted RHEAs, corresponding to the results in Figure 5.

Alloy		Concentration (at.%)			
		W	Ta	V	Zr
W5	Nominal	5.000	31.666	31.667	31.667
	Actual	6.37	34.13	29.10	30.37
	DR	10.89	55.51	26.56	7.06
	ID	2.10	13.54	34.26	50.14
W10	Nominal	10.000	30.000	30.000	30.000
	Actual	11.03	31.87	28.33	28.87
	DR	21.81	53.33	19.81	5.05
	ID	2.53	13.30	37.55	46.63
W15	Nominal	15.00	28.333	28.334	28.334
	Actual	16.15	30.65	25.90	27.30
	DR	31.20	47.07	17.44	4.27
	ID	3.34	13.43	30.96	52.25
	FDR	7.39	25.46	43.17	24.00
	FID	2.14	7.21	10.03	80.61
W20	Nominal	20.00	26.666	26.667	26.667
	Actual	21.33	29.33	24.20	25.13
	DR	33.99	42.74	19.70	3.57
	ID	3.04	9.51	24.16	63.31
W25	Nominal	25.000	25.000	25.000	25.000
	Actual	26.80	27.90	21.86	23.43
	DR	41.47	37.96	17.74	2.83
	ID	3.41	9.53	25.69	61.34

**Table 5:** The compressive mechanical properties of yield strength ($\sigma_{0.2}$ ), maximum strength ($\sigma_{m}$ ), plastic strain ($\varepsilon_{p}$ ), Vickers hardness (*HV*), and density (*ρ*) for the prepared RHEAs.

Alloy	$\sigma_{0.2}$ (Mpa)	$\sigma_{m}$ (Mpa)	$\varepsilon_{p}$ (%)	*HV* (kgf/mm^2^)	*ρ* (g/cm^3^)
W5	1679 ± 30.8	2106 ± 22.1	3.16 ± 0.2	569 ± 14.8	10.14
W10	1799 ± 65.4	2213 ± 13.0	2.45 ± 0.5	602 ± 7.4	10.56
W15	1891 ± 45.5	2231 ± 49.9	2.00 ± 0.2	615 ± 12.9	10.99
W20	1985 ± 45.0	2270 ± 52.4	1.54 ± 0.6	636 ± 8.5	11.42
W25	1934 ± 33.8	2116 ± 40.4	1.19 ± 0.4	649 ± 6.9	11.85

## Data Availability

The raw/processed data will be made available on request.
